# Mitochondrial DNA abnormalities and metabolic syndrome

**DOI:** 10.3389/fcell.2023.1153174

**Published:** 2023-03-10

**Authors:** Xudong Ding, Tingting Fang, Xiaoqi Pang, Xueru Pan, Aiying Tong, Ziyi Lin, Shikuan Zheng, Ningning Zheng

**Affiliations:** ^1^ Department of Anesthesiology, Shengjing Hospital, China Medical University, Liaoning, China; ^2^ Department of Pathophysiology, College of Basic Medical Science, China Medical University, Liaoning, China; ^3^ Shengjing Hospital, China Medical University, Liaoning, China; ^4^ Pharmaceutical Sciences, China Medical University-The Queen’s University of Belfast Joint College, China Medical University, Liaoning, China

**Keywords:** metabolic syndrome, mitochondrial copy number, mitochondrial gene mutations, mitochondrial-encoded proteins, mitochondrial proteases, mitochondrial dynamics

## Abstract

Metabolic syndrome (MetS) is a complex pathological condition that involves disrupted carbohydrate, protein, and fat metabolism in the human body, and is a major risk factor for several chronic diseases, including diabetes, cardiovascular disease, and cerebrovascular disease. While the exact pathogenesis of metabolic syndrome is not yet fully understood, there is increasing evidence linking mitochondrial dysfunction, which is closely related to the mitochondrial genome and mitochondrial dynamics, to the development of this condition. Recent advancements in genetic sequencing technologies have allowed for more accurate detection of mtDNA mutations and other mitochondrial abnormalities, leading to earlier diagnosis and intervention in patients with metabolic syndrome. Additionally, the identification of specific mechanisms by which reduced mtDNA copy number and gene mutations, as well as abnormalities in mtDNA-encoded proteins and mitochondrial dynamics, contribute to metabolic syndrome may promote the development of novel therapeutic targets and interventions, such as the restoration of mitochondrial function through the targeting of specific mitochondrial defects. Additionally, advancements in genetic sequencing technologies may allow for more accurate detection of mtDNA mutations and other mitochondrial abnormalities, leading to earlier diagnosis and intervention in patients with MetS. Therefore, strategies to promote the restoration of mitochondrial function by addressing these defects may offer new options for treating MetS. This review provides an overview of the research progress and significance of mitochondrial genome and mitochondrial dynamics in MetS.

## 1 Introduction

**FIGURE 1:**
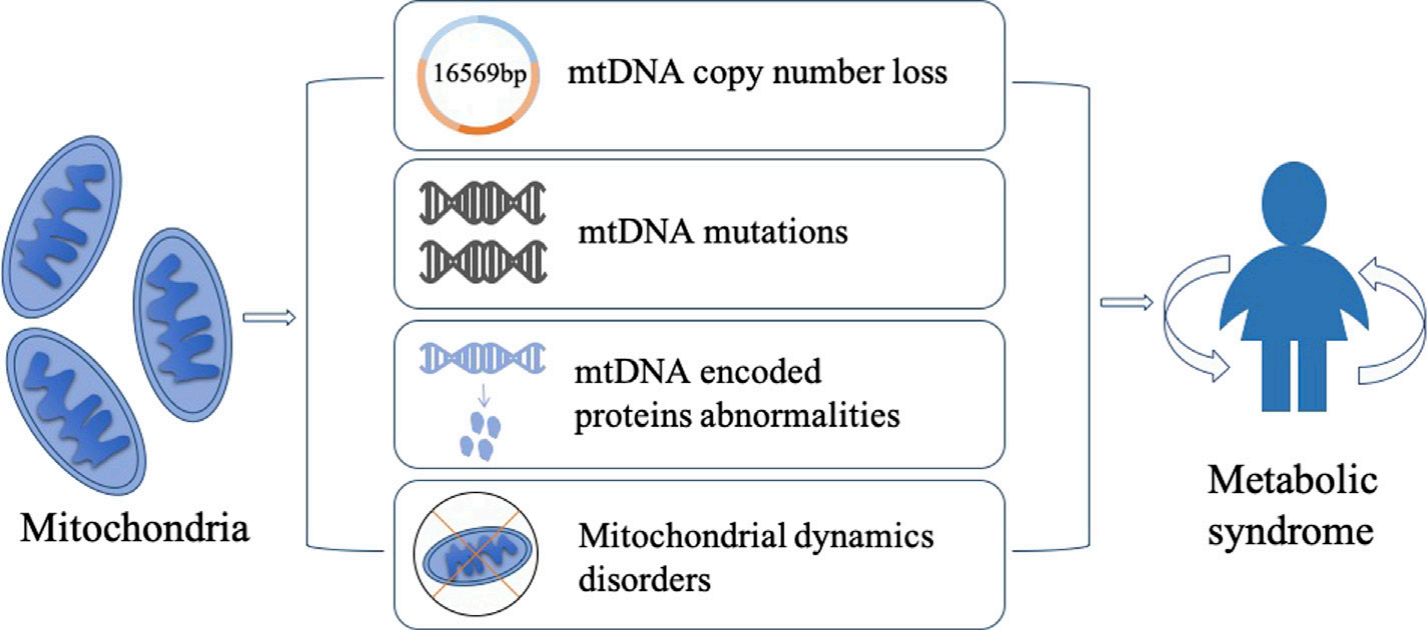
Mitochondria play a critical role in the development of metabolic syndrome, and several mechanisms have been proposed to explain their involvement. Firstly, low mtDNA copy number can lead to reduced genomic stability, potentially contributing to the pathogenesis of MetS. Secondly, mitochondria gene mutations can lead to genetic metabolic abnormalities, which is commonly observed in MetS patients. Thirdly, abnormal expression of mtDNA-encoded proteins can impair mitochondrial function and contribute to the development of MetS. Lastly, disruptions in mitochondrial dynamics, which is driven by the interplay between fusion and fission, can contribute to the accumulation of mtDNA mutations and other mitochondrial abnormalities, thereby leading to metabolic diseases. These mechanisms provide potential targets for developing novel therapeutic strategies for MetS.

Metabolic syndrome (MetS) is a complex and multifactorial disorder that affects a significant portion of the global population. It is characterized by a cluster of interrelated conditions, including hypertension, obesity, insulin resistance, dyslipidemia, and hyperglycemia, which increase the risk of cardiovascular disease, stroke, and type 2 diabetes. Although the exact etiology of MetS is not fully understood, recent research has shed light on the critical role of mitochondrial DNA (mtDNA) in the pathogenesis of MetS. Mitochondria are double-membrane organelles that play a critical role in energy metabolism, oxidative stress, and apoptosis. Mitochondrial diseases can impact a variety of cells and organs, resulting in a wide range of symptoms (Gorman et al., 2016). Mitochondria have their own DNA, which is distinct from nuclear DNA (nDNA). The different structural and functional properties of mtDNA and nDNA lead to their different applications in science. With its significantly higher mutation rate, mtDNA has been used as a powerful tool to trace the lineage. Methods have been developed to trace the ancestry of many species over hundreds of generations and have become a mainstay of phylogenetic and evolutionary biology (Bernardo et al., 2014). mtDNA is a circular DNA molecule that exists in multiple copies in each mitochondrion. They encode a small number of genes involved in oxidative phosphorylation. mtDNA abnormalities, such as mutations, deletions, copy number loss, and rearrangements, can disrupt the energy production capacity of the mitochondria, leading to the generation of excess reactive oxygen species (ROS), which can cause oxidative damage to DNA, proteins, and lipids. Recent studies have shown that mtDNA copy number loss and mutations in mtDNA-encoded proteins are associated with insulin resistance and glucose intolerance. Moreover, mtDNA abnormalities can lead to increased oxidative stress, impaired mitochondrial function, and altered energy metabolism, all of which are thought to be involved in the pathophysiology of MetS. Additionally, disturbances in mitochondrial dynamics, such as changes in mitochondrial fission and fusion, have been linked to the development of insulin resistance and obesity. Here we will summarize the current state of knowledge on the relationship between mtDNA abnormalities and MetS, with a focus on mtDNA copy number loss, mutations, abnormalities of mtDNA-encoded proteins, and mitochondrial dynamics imbalance. The purpose of this review is to provide a comprehensible overview of the role of mtDNA in MetS and to identify potential therapeutic targets for the prevention and treatment of this increasingly prevalent disease (Figure 1).

## 2 mtDNA copy number loss in metabolic syndrome

Nuclear gene mutations that lower mtDNA expression or primary mtDNA mutations that directly impairs the function or quantity of the gene products encoded by mtDNA cause mitochondrial disorders (Gorman et al., 2016; Filograna et al., 2021). MetS is a collection of metabolic abnormalities that may be linked to variations in mitochondrial DNA content (Samson and Garber, 2014). In healthy humans, there are usually hundreds to thousands of copies of mtDNA in each cell. The number of mtDNA copies can vary between cell types and tissues. In MetS patients, the number of mtDNA copies has been found to be lower compared to healthy individuals. Specifically, a number of studies have reported that MetS patients have reduced mtDNA copy number in their adipose tissue, skeletal muscle, and blood cells. The degree of reduction can vary, but in general, tend to have a lower mtDNA copy number. In leukocytes, the MetS group has a lower mtDNA copy number than the non-MetS group, with a lower mtDNA copy number linked to low plasma high density lipoprotein (HDL) and high triglycerides (Huang et al., 2011). The number of copies can be utilized to assess mitochondrial dysfunction (Fazzini et al., 2021). The difference in mtDNA copy number before and after disease suggests that mtDNA plays a regulatory function in the development of a variety of chronic diseases, including cardiovascular disease, kidney disease, liver disease, neurological disease, and cancer, all of which have therapeutic potential (Castellani et al., 2020).

mtDNA copy number was found to be inversely related to metabolic syndrome and type 2 diabetes risk. Obesity characteristics mediate a large percentage of the overall effect of mtDNA copy number on type 2 diabetes (Fazzini et al., 2021). Human mitochondrial DNA haplogroups are genetic haplogroups defined by mitochondrial DNA differences (Swerdlow et al., 2020). In diverse populations, mtDNA haplogroups are hypothesized to be associated in a variety of metabolic illnesses, including metabolic syndrome, obesity, T2D, and T2D-related comorbidities. The N9a haplogroup is a subgroup of the mtDNA N9 haplogroup, which is commonly found in East Asia and Southeast Asia. The experiment confirmed that mtDNA haplogroups N9a (N9a1 and N9a10a) exhibit lower activity of the respiratory chain complex compared to three non-N9A haplogroups (D4j, G3a2, and Y1). This difference could contribute to changes in mitochondrial function and REDOX status. The mtDNA haplogroup plays a critical role in mitochondrial function and mitochondria-mediated signaling pathways and has been associated with the development of T2D. Insulin resistance, a hallmark of T2D, may result from reduced oxidative phosphorylation, which increases the generation of reactive oxygen species (ROS), which is a key regulator of T2D-related insulin receptor signaling and inflammation. The mitochondrial retrograde signaling pathways of two N9a hybrids and three non- N9A hybrids were observed. The effect of this retrograde signaling change on diabetes in cellular models was further studied (Hua et al., 2019). The experiment revealed the mechanism of N9a in T2D and established the mitochondrial retrograde signaling pathway. The presence of N9a in T2D was confirmed by other evidence: most of the observed changes in biological characteristics were related to metabolic regulation. Another study suggested that N9a haplogroup may not be a protective factor for T2D (Hwang et al., 2011). Hezhi Fang showed that the mitochondrial REDOX signaling pathway, which is usually associated with insulin sensitivity, drove ERK1/2 phosphorylation/activation and led to changes and activation of insulin-stimulated glucose uptake in N9a and non-N9A cells by targeting the underlying signaling pathway (Fang et al., 2018). The gene TLR4 on chromosome nine exhibited an increased response to ERK1/2 overactivation in N9a cells but reduced insulin-stimulated glucose uptake (Fang et al., 2018). Steatohepatitis-associated circRNA ATP5B Regulator (SCAR), which is located in mitochondria, inhibits mitochondrial ROS (mROS) output and fibroblast activation (Zhao et al., 2020). The relationship between mtDNA copy number and mitochondrial function was confirmed in knockdown cell models with reduced mtDNA copy number, which resulted in reduced expression of important complex proteins, changes in cell morphology, and decreased activity of respiratory enzymes. The models showed that mitochondrial function could be restored by bringing the mtDNA copy number back to wild-type levels. When mtDNA copy number levels decreased, they can increase the production of ROS and lead to oxidative stress, which is linked to a decline in mitochondrial function (Castellani et al., 2020).

mtDNA levels depend on the stability of the mitochondrial genome through appropriate mitochondrial translation. Mitochondrial ribosomal proteins (MRPs) are the key components responsible for the translation of mitochondrial gene coding proteins. Abnormal structure and function of MRPs will affect the synthesis of mitochondrial coding proteins, reduce mtDNA copy number and the efficiency of oxidative phosphorylation, and finally lead to metabolic disorders. With the discovery of mtDNA mutations, the genotypes, clinical symptoms and diagnosis and treatment of mitochondrial diseases have been rapidly developed, especially the relationship between mtDNA copy number and clinical diseases has been paid more and more attention. Changes in the copy number of mitochondrial DNA indicate the onset of metabolic syndrome and other diseases, it provides convenient conditions for the research and treatment of diseases. It is important to note that mtDNA copy number alone is not a definitive diagnostic tool for metabolic syndrome, and its use in clinical practice is still limited. However, it can serve as a useful biomarker for assessing mitochondrial dysfunction and metabolic disease risk in certain populations. Further research is needed to better understand the relationship between mtDNA copy number, metabolic dysfunction, and potential therapeutic interventions.

## 3 Mitochondrial gene mutations and genetic metabolic abnormalities

Mitochondrial diseases are multisystem diseases with oxidative phosphorylation defects and great clinical, biochemical and genetic heterogeneity. The distribution and relative level of heterogeneity of mtDNA mutations may lead to different rates of aging and disease progression between individual cells (Hahn and Zuryn, 2019). Endocrine dysfunction is often observed in hereditary mitochondrial diseases, reflecting reduced intracellular hormone production or extracellular secretion. Diabetes is the most frequently described endocrine disorder in patients with inherited mitochondrial diseases, but other endocrine manifestations in these patients may include growth hormone deficiency, hypogonadism, adrenal dysfunction, hypoparathyroidism, and thyroid disease. Although mitochondrial endocrine dysfunction often occurs in the context of multisystem diseases, some mitochondrial diseases are characterized by isolated endocrine involvement (Chow et al., 2017). While a single gene mutation can play a role in mediating obesity, it is often the result of the interaction of genetic and environmental factors. To date, nuclear gene mutations related to obesity, such as MC4R, BDNF, FTO, etc., have been identified (McCaffery et al., 2012). However, the association between mtDNA mutations and childhood obesity remains to be elucidated. Several mutations in the mitochondrial tRNA gene have been found to be associated with diabetes and metabolic disorders. It suggests that mtDNA mutation may be related to obesity. For example, tRNA thr mutations C (10003T > C), and tRNA Glu mutations C (14709T > C) and G (14692A > G) are associated with such diseases (Wang et al., 2020). The point mutation of 3243rd nucleotides in mitochondrial tRNA-Leu (UUR) can cause maternal diabetes and deafness (Alves et al., 2017). Mitochondrial diabetes is caused by mutations in mitochondrial genes. β Diabetes caused by oxidative phosphorylation is a rare type of diabetes. Mitochondrial tRNALeu (UUR) 3243A > G-spot mutation is the most common cause of mitochondrial diabetes (Capriglia et al., 2021). Kearns-Sayre syndrome, which is caused by a large deletion in the mitochondrial DNA, can also be associated with diabetes (Kamal et al., 2015) (Table 1).

**TABLE 1:** Mitochondrial gene mutation-associated Mitochondrial syndrome.

Mitochondrial syndrome	Mutant mitochondrial genes
Obesity	tRNA thr mutations m.10003t > C
	tRNA Glu mutations m.14709t > C and m.14692a > G
Maternal diabetes and deafness	3243rd nucleotides in mitochondrial tRNA-Leu (UUR)
Mitochondrial diabetes	Mitochondrial tRNALeu (UUR) 3243A > G-spot

mtDNA is different from nuclear DNA and is not protected by histones. As a result, it is more susceptible to the attack of mitochondrial ROS and is prone to mutation, which may be accelerated in diabetes. It has been suggested that increased ROS production (OH-, H_2_O_2_) may be the core of diabetic pathology during hyperglycemia (Li et al., 2008). The characteristics of mitochondrial diabetes, also known as maternally inherited diabetes and deafness (MIDD), include maternal inheritance of diabetes, nerve deafness, relatively low body weight (BMI), early onset age (between 30 and 40 years), progressive decline in islet cell function leading to insulin-dependent diabetes mellitus, negative urine sugar antibody, other neuromuscular lesions, and progressive sensorineural hearing loss (Tsang et al., 2018). The premature death of MIDD patients due to cardiac causes is a significant problem. It is reported that in some of these cases, the mutation load in myocardium is more abundant than that in blood (Yoshida et al., 1994), indicating that the reduction of cardiac ATP synthesis is the most likely stimulator of cardiomyocyte hypertrophy and failure in these individuals. Other systemic manifestations of MIDD patients: MELAS syndrome (mitochondrial myopathy, encephalopathy with lactic acidosis and recurrent stroke like attack). MELAS should be considered as a clinical diagnosis when young MIDD patients have a stroke. Lactic acidosis is caused by the decrease of blood pH and buffer capacity due to the increase of lactic acid concentration. In MELAS patients, abnormal mitochondria can not metabolize pyruvate, resulting in a large amount of pyruvate to produce lactic acid, which accumulates in blood and body fluids. A characteristic pathological change of MELAS patients is the accumulation of a large number of abnormal mitochondria in the arterioles and capillary walls of brain and muscle. It is reported that 13% of the 199 affected members of 45 families with m.3243A > G mutation have a combination of MIDD and MELAS characteristics, and deafness is the only manifestation in some patients (Gerbitz et al., 1995). The reduced uptake of brain single photon emission computed tomography (SPECT), especially in the bilateral parietooccipital or occipital regions in MIDD (Suzuki et al., 1996), may reflect the low metabolic state of these neurons due to mitochondrial respiratory dysfunction (Murphy et al., 2008). The study shows that the occurrence of diabetic complications depends not only on glycemic control, but also on individual factors that may be related to genetic heterogeneity (Alves et al., 2017) Specifically, mtDNA is almost completely composed of coding regions, which is easy to be damaged due to the lack of histone protection, and lacks an effective self-healing mechanism. Its mutation frequency is significantly higher than that of nuclear genes. The 3243A to G-spot mutation may be one of the genetic factors leading to familial aggregation of diabetes (Li et al., 2008). A Rötig found that almost 3/4 of the mtDNA mutation patients had family history of diabetes, almost all related to the maternal mitochondrial genetic pattern (Rötig et al., 1996). Recent studies have shown that individuals may have more than two point mutations, which could contribute to the pathogenesis of diabetes. However, Rötig’s team did not identify any subjects with multiple mutations, possibly due to limitations in the technology used for analysis, which was not based on direct DNA sequencing. While these alternative technologies may be clinically convenient, they may not be the most sensitive for detecting point mutations (Li et al., 2008).

## 4 mtDNA-encoded proteins and metabolic syndrome

**FIGURE 2:**
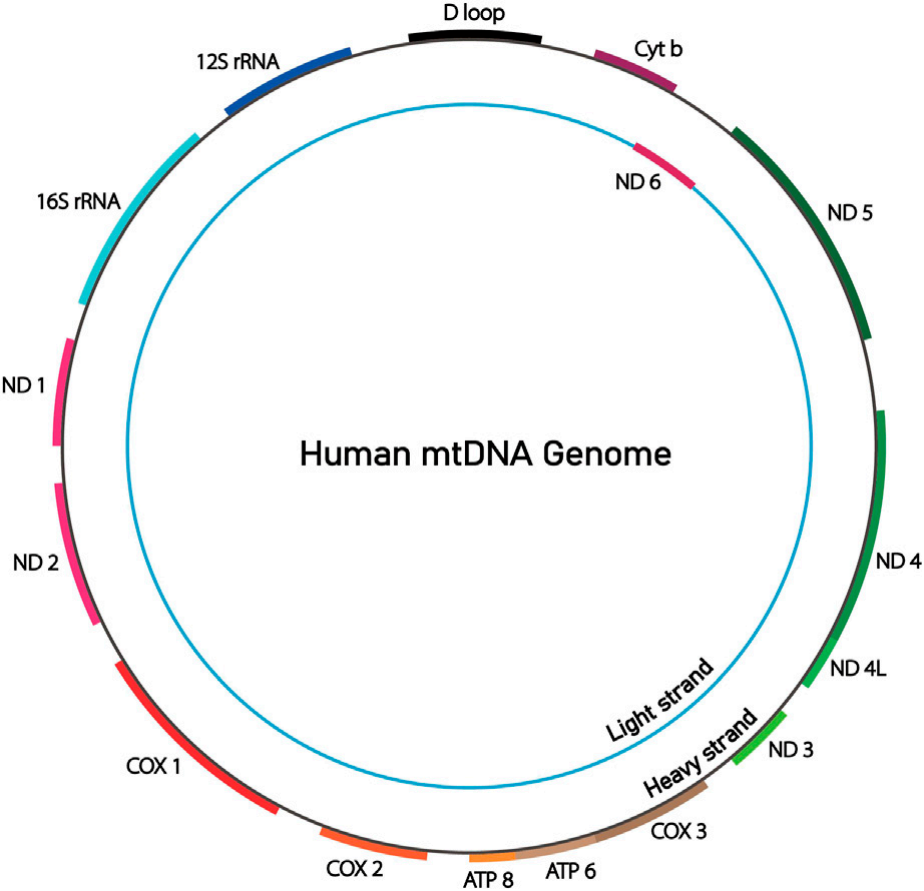
This is a diagram of the mitochondrial genome. It encodes for 13 essential proteins involved in respiratory chain electron transfer and oxidative phosphorylation. ND1, ND2, ND3, ND4, ND4L, ND5, ND6 are subunits of complexI. Cyt b is subunit of complexIII. COX1, COX2, COX3 are subunits of complexIV. ATP8 and ATP6 are subunits of complexV. It also encodes for 22 transfer RNAs (tRNAs) and 2 ribosomal RNAs (rRNAs) that are necessary for protein synthesis within the mitochondria.

There are some significantly differences between the structure of mtDNA and nDNA, instead mtDNA is similar to bacterial chromosomes. The structure of mtDNA molecule is a double-stranded closed circular DNA molecule including a heavy outer ring (H) and a light inner ring (L) with no intron sequences except for a small region related to mtDNA replication and transcription. There are 13 proteins encoded by mtDNA associated with metabolic syndrome, they are the core subunit of the oxidative phosphorylation (OXPHOS) complexes I, III, IV, and V. E.g., seven subunits (ND 1–6 and 4L) of complex I, cytochrome b (Cyt b) of complex III, the COX I–III subunits of cytochrome oxidase or complex IV, and the ATPase six and 8 subunits of ATP synthase (Figure 2). Known so far, Metabolic syndrome associated with proteins encoded by mtDNA include: T2DM, CMP, MELAS, hypertension, and hyperlipidemia, etc., (Sharma and Sampath, 2019; Finsterer, 2020).

The increased risk of cardiovascular disease is an important cause of diabetes (Fiorentino et al., 2013). All mitochondrial genomes of the maternal relatives of T2DM subjects aged 28–71 years with an average age of 43 years were screened by PCR-Sanger sequencing. Analysis and research found that both families have ND1 T3394C mutations, as well as multiple sets of mutations in Y2 and M9a in the mitochondrial haplogroup. The m.T3394C mutation on the tyrosine at position 30 of ND1 may lead to the failure of ND1 mRNA metabolism, leading to mitochondrial dysfunction. The m.A14693G mutation in the TΨC-loop of tRNAGlu is critical to the formation and stability of the tRNA structure, its mutation may cause tRNA metabolism disorder, thereby aggravating the mitochondrial dysfunction caused by the ND1 T3394C mutation (You et al., 2022). There is another group of studies on the clinical, genetic and biochemical characteristics of the maternal genetic lineage of T2DM in China. Through PCR and direct sequencing analysis, it was found that there are two potential pathogenic mutations in ND1 and ND2. The level of reactive oxygen species in T2DM patients with both m.T4216C and m.C5178A mutations was significantly increased (*p* < 0.05). In addition, plasma levels of malondialdehyde and 8- hydroxydeoxyguanosine in patients with T2DM were significantly increased, and the level of superoxide dismutase was decreased (*p* < 0.05). In summary, ND1 T4216C and ND2 C5178A mutations may cause oxidative stress, impair mitochondrial function, and cause the pathogenesis of T2DM (Jiang et al., 2021).

Studies have shown that increased production of reactive oxygen species may be the cause of obesity and hyperglycemia. The experiment found that four new mutations were found in the mitochondrial genes of ND1, ATPase 8, ND5 and Cyt b of the subject: W121 (1)G (3667T>G), M42 (2)T (8490T>C), V290 (1)I (13204G>A) and V170 (3)V (15256A>G). Using silicon analysis to analyze mutations in the structure and function of these proteins. The study found that the mutation of ATPase 8 (T8490C) neither changed its secondary structure nor its function. Because the new suspected pathogenic mutation was not present in 105 race-matched controls, ATPase 8 is not a causative factor. However, the synergistic activity of Cyt b, ATPase 8, ND1 and ND5 genes may be a factor of secondary complications in patients with chronic T2D. It is also the cause of hypertension and hyperlipidemia (Elango et al., 2014). Studies induced chronic T2DM (cT2DM) model by intraperitoneal injection of STZ (35 mg/kg) after 6 weeks of a high-fat and high-sugar diet. H&E staining was used to observe the morphological damage of rat hippocampus. The expression of inflammatory mediators (COX-2, TNF-α, IL-1β) and oxidative stress indicators (MDA, p22phox) in the brain tissue of cT2DM rats is increased, and studies have evaluated homozygous, heterozygous, dominant and recessive models, COX2 in 3′-UTR may contribute to the etiology of T2DM or adjust its risk. Therefore, increased COX2 may be associated with T2DM (Ozbayer et al., 2018; Xie et al., 2018). The last group of through direct sequencing of PCR fragments of a 14-year-old index male hypertrophic cardiomyopathy (hCMP) patient, it was found that m.14757T> a, m.15236A>G, m.15314G> a resulted in amino acid residues in the Cyt b gene replace, Cyt b is very likely to be related to cardiomyopathy (Zarrouk-Mahjoub et al., 2015). The 3697G>A mutation can cause to isolated severe complex I defects, leading to lactic acidosis, central nervous system dysfunction and other metabolic syndromes (Zhong et al., 2019). Modification of proteins encoded by mtDNA is still a technical bottleneck at present. It is expected that the continuous progress of mitochondrial gene editing technology can provide a new breakthrough for the treatment of metabolic diseases by targeting mtDNA encoding proteins.

## 5 Mitochondrial dynamics drived mtDNA change and metabolic diseases

The process of mitochondrial fusion, fission, biogenesis, and mitophagy that determines mitochondrial morphology, quality, and abundance are known as mitochondrial dynamics (Vásquez-Trincado et al., 2016). Fusion and fission processes are essential for the maintenance of important cellular functions such as mitochondrial respiratory activity, mitochondrial DNA (mtDNA) distribution, apoptosis, cell survival or calcium signalling. ATP production is modulated by mitochondrial networks generated by fusion and this pathway is controlled by transmitting the membrane potential from areas of high O2 availability to those with low availability, thus allowing the dissipation of energy (Westermann, 2010). The selective elimination of dysfunctional mitochondria, a quality-control process that guarantees a healthy mitochondrial population, is a dynamic characteristic of mitochondria (Chan, 2020). Thus, many dynamic properties of mitochondria maintain cell function, one of which is to influence the amount and distribution of mtDNA to cause many diseases.

The mutants of mtDNA caused mitochondrial illnesses are usually recessive, and the mutational burden must reach high levels, perhaps 60–90 percent heteroplasmy, before cells exhibit respiratory chain dysfunction. In skeletal muscle, mutant mice with the mitochondrial fusion genes Mfn1 and Mfn2 disrupted show substantial mtDNA depletion that precedes physiological problems such as the mutant mouse display low blood glucose levels under fasting conditions and reduced body temperatures. Furthermore, the mutant muscle’s mitochondrial genomes rapidly accumulate point mutations and deletions. In a separate investigation, we discovered that disrupting mitochondrial fusion significantly enhances mitochondrial dysfunction and death in a mouse model with high mtDNA mutation levels. Mitochondrial fusion is likely to be a protective factor in human illnesses linked with mtDNA mutations due to its dual function of maintaining mtDNA integrity and retaining mtDNA function in the face of mutations (Chen et al., 2010). When cells are exposed to a substantial nutrition supply, such as in obesity or type 2 diabetes, this pro-fusion condition is prevalent in instances of enhanced energy efficiency owing to famine or acute stress. Excess nutrition exposure enhances mitochondrial fission and inhibits mitochondrial fusion, both of which are linked to uncoupled respiration (Liesa and Shirihai, 2013). Type 2 diabetes has been linked to a decrease in the expression of OXPHOS-CR involved in mitochondrial biogenesis and oxidative phosphorylation activity (Mootha et al., 2003). When obese patients were compared to lean co-twins, it was discovered that they had a global expressional downregulation of mitochondrial oxidative pathways, as well as a concomitant downregulation of mtDNA, mtDNA-dependent translation system, and protein levels of the oxidative phosphorylation machinery. In fact, fatty acid oxidation, ketone body formation and breakdown, and the tricarboxylic acid cycle were shown to be negatively linked with insulin resistance, obesity, and inflammatory cytokines, indicating a downshift (Heinonen et al., 2015). Furthermore, Mitochondrial biogenesis helps to regulate energy balance, and increased ROS produced from electron transport chain under hyperglycemic conditions is thought to exacerbate pathological pathways, leading to diabetic microvascular and macrovascular complications (Brownlee, 2001).Mitochondrial kinetics and functional disorders are closely related to myocardial contractile dysfunction and myocardial injury. Fusion is a process that takes place under the tight control of mitofusion1 (Mfn1), mitofusion2 (Mfn2), and Opa1. After mitochondrial fusion, the possibility of being degraded is reduced. Fission is regulated by a series of signaling molecules such as Dynamin-related protein 1 (Drp1) and mitochondrial fission factor (Mff), a process that is primarily aimed at removing damaged mitochondria. Fission isolates damaged fragments of mitochondria and induces mitophagy. Imbalance of myocardial mitochondrial dynamics and homeostasis is one of the pathophysiological processes underlying the numerous cardiac symptoms of the metabolic syndrome. The mechanisms of mitochondrial dynamic have lately been linked to cardiovascular disease. Such as changes in mitochondrial dynamics play a key role in cardiovascular remodeling. Cardiac hypertrophy, diabetic cardiomyopathy, myocardial infarction, and atherosclerosis all have mitochondrial network fragmentation as a common pathogenic characteristic. Mitochondrial morphological changes eventually result in metabolic failure, mitochondrial DNA damage, and/or cell death (Vásquez-Trincado et al., 2016). Galloway et al. observed in diabetic cardiomyopathy that within 3 weeks of hyperglycemia, no significant changes in mitochondrial morphology occurred, but after 5 weeks of hyperglycemic environment, this was accompanied by increased proteolytic cleavage of Opa1 and impaired fusion processes leading to more mitochondrial fragmentation (Galloway and Yoon, 2015). Cells can also protect cardiomyocytes by inhibiting AMPK-based related signaling pathways, inhibiting mitochondrial division, promoting mitochondrial fusion, enhancing mitochondrial capacity, and reducing ROS damage. Research has revealed that aging-related mtDNA mutations in adult cardiac progenitor cells (CPCs) may alter receptor-mediated mitophagy in the differentiation process, resulting in persistent fission and less functioning fragmented mitochondria (Lampert et al., 2019). An Opa1 heterozygous mouse with abnormal mitochondrial morphology (mitochondria with abnormal cristae and mitochondrial tissue) showed an increase in ROS and a reduction in mitochondrial DNA content, implying compromised mitochondrial function. Mitophagy disruption caused accumulation of enlarged mitochondria in cardiac tubes and dilated cardiomyopathy in a Parkin knockout *Drosophila* model (Dorn et al., 2015). Mitochondrial dynamics directly influence pancreatic function as well. In ob/ob mice, OPA1 levels decrease in pancreatic islet cells before the onset of diabetes. Silencing Opa1 in pancreatic β cells using a Cre-loxP system yields similar results. β cells lacking OPA1 maintained normal mtDNA copy number, but the levels and activity of the electron transport chain complex IV were dramatically decreased, resulting in poor glucose stimulation of ATP generation and insulin secretion. Thus, changes in many dynamic characteristics of mitochondria can cause many diseases, such as metabolic syndrome, many of which are related to changes in mitochondrial DNA.

## 6 Conclusion

mtDNA abnormalities have emerged as a significant factor in the development of MetS. The mechanisms by which these abnormalities contribute to MetS are complex and include quantitative changes in mtDNA copy number, mutations affecting mtDNA-encoded proteins, and imbalances in mitochondrial dynamics. Evidence suggests that these abnormalities can affect key metabolic pathways, including glucose metabolism, lipid metabolism, and insulin signaling, ultimately leading to the development of metabolic syndrome. Despite the significant progress made in understanding mtDNA abnormalities and their contribution to MetS, several challenges still remain. One significant limitation is the small size of the mitochondrial genome, which can make identifying specific mutations challenging. Additionally, mtDNA mutations can occur at varying levels of heteroplasmy, which can make their effects difficult to predict. Furthermore, there is often significant variability in the presentation and progression of these disorders, which can make it difficult to establish clear cause-and-effect relationships between specific mtDNA mutations and clinical outcomes. The study of mtDNA abnormalities and MetS is of critical importance to clinical care. Identifying mtDNA abnormalities can aid in the diagnosis, prevention, and treatment of this condition. mtDNA copy number has been proposed as a biomarker for metabolic disease, and its assessment can be used to monitor disease progression and the response to therapy. Also, identifying new mtDNA mutations associated with metabolic disease can help improve the accuracy of diagnosis and identify new therapeutic targets. Based on the many different properties of mtDNA and nDNA, the current widely used gene editing technologies have many limitations in the field of mitochondrial genes. Future prospects depend largely on the development and application of mitochondrial gene-specific editing technologies, which has clearly become an urgent technical challenge.
